# SSRI-associated clinical outcomes in older adults with behavioral and psychological symptoms of dementia

**DOI:** 10.1093/geroni/igaf122.1651

**Published:** 2025-12-31

**Authors:** Chun-Ting Yang, James Wilkins, Dae Hyun Kim, Joshua Lin

**Affiliations:** Brigham and Women’s Hospital, Boston, Massachusetts, United States; McLean Hospital, Belmont, Massachusetts, United States; Hebrew SeniorLife, Boston, Massachusetts, United States; Brigham and Women’s Hospital, Boston, Massachusetts, United States

## Abstract

Citalopram, escitalopram, and sertraline are three selective serotonin reuptake inhibitors (SSRIs) that are commonly prescribed in older adults with behavioral and psychological symptoms of dementia (BPSD), but evidence about their comparative safety remains limited. This retrospective cohort study used Medicare 2013-2018 and Optum Clinformatics® 2013-2024 to examine the risks of clinical outcomes between different SSRIs in older adults with BPSD. BPSD individuals aged≥65 years and newly prescribed citalopram, escitalopram, or sertraline were included. Propensity score matching was employed to balance baseline covariates. Outcomes included all-cause mortality, all-cause hospitalization, and hospitalization reasons. The on-treatment effect was measured using Cox proportional hazards model. Analyses were conducted separately in the two databases, and the effect estimates were pooled by fixed-effect meta-analysis. A total of 15,562 citalopram-sertraline and 17,180 escitalopram-sertraline matched pairs were analyzed. Compared with sertraline, both citalopram and escitalopram showed non-significantly different risks of all-cause death (hazard ratio [95% CI]: 0.96 [0.91-1.01] for citalopram; 0.95 [0.91-1.01] for escitalopram), inpatient delirium (1.05 [0.97-1.15]; 1.03 [0.95-1.11]), falls (1.04 [0.94-1.16]; 1.04 [0.94-1.14]), stroke (1.05 [0.88-1.26]; 1.13 [0.95-1.33]), ventricular arrhythmia (0.63 [0.21-1.94]; 1.00 [0.39-2.53]), and hyponatremia (1.00 [0.87-1.16]; 1.02 [0.90-1.17]). A marginally increased risk of all-cause hospitalization was observed in citalopram users versus sertraline users (1.05 [1.01-1.09]), but this increase was not statistically significant for escitalopram users (1.05 [1.00-1.09]). The modest magnitude of the HR estimate, along with the marginal significance, may suggest limited clinical relevance. Our findings suggest the comparable safety profiles between the use of citalopram, escitalopram, and sertraline in older adults with BPSD.

